# Meagre *Argyrosomus regius* (Asso, 1801) Stem Spermatogonia: Histological Characterization, Immunostaining, In Vitro Proliferation, and Cryopreservation

**DOI:** 10.3390/ani10050851

**Published:** 2020-05-14

**Authors:** Rosa Zupa, Nicola A. Martino, Giuseppina Marzano, Maria E. Dell’Aquila, Aldo Corriero

**Affiliations:** 1 Department of Emergency and Organ Transplantation, Section of Veterinary Clinics and Animal Production, University of Bari Aldo Moro, 70100 Valenzano, Italy; aldo.corriero@uniba.it; 2 Department of Veterinary Science, University of Turin, 10124 Turin, Italy; nicolaantonio.martino@unito.it; 3 Department of Biosciences, Biotechnologies and Biopharmaceuticals, University of Bari Aldo Moro, 70126 Bari, Italy; giuseppina.marzano@unisalento.it (G.M.); mariaelena.dellaquila@uniba.it (M.E.D.)

**Keywords:** stem spermatogonia, fish reproduction, germ cell characterization, gem cell culture

## Abstract

**Simple Summary:**

The meagre, *Argyrosomus regius*, is a commercial fish species of which aquaculture production is limited due to technological bottlenecks such as inconstant reproduction in captivity. In addition, there are only a few broodstocks in European farms, which rises the problem of limited genetic variability of the cultured meagre population. The development of germ cell xenotransplantation technology might increase aquaculture production through the production of fertile meagre gametes in a host species for which farming technology is well established. Spermatogonial stem cells have the capacity to proliferate and to proceed through the spermatogenesis process, eventually giving rise to mature spermatozoa. In the present study, meagre spermatogonial stem germ cells were identified, isolated, cultured in vitro, and cryopreserved. Isolated spermatogonial stem cells proliferated, maintained their stem properties in culture, and were still viable after cryopreservation and thawing. This work represents a first step towards the development of a xenotransplantation technology that might facilitate the production of this valuable species in captivity.

**Abstract:**

The meagre, *Argyrosomus regius*, is a valued fish species of which aquaculture production might be supported by the development of a stem germ cell xenotransplantation technology. Meagre males were sampled at a fish farm in the Ionian Sea (Italy) at the beginning and end of the reproductive season. Small and large Type A undifferentiated spermatogonia were histologically identified in the germinal epithelium. Among the tested stemness markers, anti-oct4 and anti-vasa antibodies labeled cells likely corresponding to the small single Type A spermatogonia; no labeling was obtained with anti-GFRA1 and anti-Nanos2 antibodies. Two types of single A spermatogonia were purified via density gradient centrifugation of enzymatically digested testes. Testes from fish in active spermatogenesis resulted in a more efficient spermatogonial stem cell (SSC) yield. After cell seeding, meagre SSCs showed active proliferation from Day 7 to Day 21 and were cultured up to Day 41. After cryopreservation in dimethyl-sulfoxide-based medium, cell viability was 28.5%. In conclusion, these results indicated that meagre SSCs could be isolated, characterized, cultured in vitro, successfully cryopreserved, and used after thawing. This is a first step towards the development of a xenotransplantation technology that might facilitate the reproduction of this valuable species in captivity.

## 1. Introduction

The meagre, *Argyrosomus regius* (Asso, 1801), is a member of the Sciaenidae family, mostly found near estuaries of the Mediterranean Sea and the Atlantic coast of Europe and North Africa [1,2,3]. Its flesh has a high quality due to its very high content of polyunsaturated fatty acids, low fat content, excellent taste, firm texture, and long shelf-life [4,5]. Meagre fishery production in Europe has historically been quite small [6], and a decline of the European fishery during 1984–2002 has contributed to the creation of lucrative European niche markets in which this species is priced in the middle range of 6–12 Euro/kg [5,7]. Consumer appreciation and the good market value have stimulated the interest of the aquaculture sector in this species, which was included in the group of new/emerging fish species investigated as aquaculture candidates within the EU FP7 project ‘Exploring the biological and socio-economic potential of new/emerging candidate fish species for the expansion of the European aquaculture industry’ (DIVERSIFY) [8] and the EU H2020 project ‘New technologies Tools and Strategies for a Sustainable, Resilient and Innovative European Aquaculture’ (NewTechAqua) [9].

According to the available literature, wild meagre females attain sexual maturity at a total length of 47–70 cm and males at 45–62 cm [10,11]. The reproductive season of meagre wild populations extends from March to July–August in the eastern Atlantic [11,12] and in the Mediterranean [10]. Hatchery-produced meagre have a limited capacity to complete gametogenesis and spawn spontaneously [13], and hormonal treatments are required to induce spawning [7,14,15]. Fertilized eggs obtained through hormonal induction of gamete maturation and spawning have been used to set up rearing protocols in south-eastern Atlantic (Canary Islands) and Mediterranean countries [7]. Nowadays, European meagre aquaculture production is mainly concentrated in Spain, with smaller quantities produced in France, Portugal, Italy, Greece, and Croatia [16]. The meagre market is now slowly expanding, and the improvement of rearing technologies in order to reduce production costs could stimulate farmers’ interest in this species and support a faster market expansion [7]. One of the major bottlenecks that needs to be overcome in order to expand meagre aquaculture production is the limited genetic variability of the existing broodstocks in Europe, which have originated from only three different wild stocks [17].

In recent years, in addition to the traditional breeding protocols, innovative biotechnologies have begun to emerge in aquaculture. Among these biotechnologies, stem germ cell xenotransplantation (i.e., the isolation of a stem germ cell from a donor animal and its subsequent transplantation into an infertile recipient) could be also successfully applied to aquaculture breeding programs [18,19,20,21]. Xenotransplantation would offer the advantage of using wild meagre caught by commercial fisheries as donors of stem germ cells able to differentiate in mature germ cells in a recipient species with reproduction that is easily controlled in farming conditions. To date, significant and encouraging studies have been conducted on fish germ cell transplantation, in which primordial germ cells, spermatogonial stem cells (SSCs), and oogonia were transplanted into embryos, larvae, or adult fish of a host species [19,22,23,24,25,26]. SSCs are the most undifferentiated cells of spermatogenesis, and have the potential for both self-renewal and differentiation [27]. SSC identification, isolation, and in vitro proliferation are crucial preliminary steps needed to set up an efficient spermatogonial xenotransplantation technique [22]. However, the identification of SSCs in fish is problematic due to the few available molecular markers, the expression of which shows high variability in the different species [28,29,30].

The aim of the present study was to identify and characterize SSCs in adult meagre and to establish suitable conditions for their in vitro proliferation and cryopreservation.

## 2. Materials and Methods

### 2.1. Ethical Statement

The present study was carried out on testis sampled from dead fish harvested from the Rheomare fish farm (Gulf of Tarentum, North Ionian Sea, Italy). Fish were harvested for commercial purposes according to routine farming procedures, and then purchased and sampled. Ethical approval was not required because the study did not involve handling of live animals.

### 2.2. Fish Sampling

For the present study, four (n = 2 per trial) hatchery-produced adult meagre males reared in sea cages were sampled in mid-July 2017 (Trial 1) and mid-March 2018 (Trial 2). Fish were harvested according to routine commercial farm procedures and promptly transported by refrigerated van to the fish-processing facility. During Trial 1, in order to identify fish sex, light abdominal pressure was applied to stimulate sperm release in males. Since fish sampled in Trial 2 did not release sperm after abdominal pressure, their sex was identified by microscopic observation of a postmortem gonadal biopsy sample. The selected fish (Trial 1: mean total length = 56.2 ± 6.7 cm; Trial 2: mean total length = 59.2 ± 1.7 cm) were transported on crushed ice to the laboratory, where they arrived within 2 h.

### 2.3. Histological Analysis of Meagre Testis and Immunohistochemical Localization of Spermatogonial Stem Cells

Testis cross sections (0.5 cm tick) were taken from each fish and fixed in 10% buffered formalin, 4% paraformaldehyde in phosphate-buffered saline (PBS) (Sigma-Aldrich S.r.l., Milan, Italy) or Bouin’s solution; dehydrated in ethanol; clarified in xylene; and embedded in paraffin wax.

For basic histological analysis, only samples fixed in Bouin’s solution were used [31]. For this purpose, 4 μm thick sections were cut and stained with haematoxylin–eosin (H-E). The different germ cell types and the spermatogenetic phase were identified according to Zupa et al.’s (2017) [32,33] procedure for greater amberjack *Seriola dumerili.*

For immunohistochemical identification of meagre SSCs, the following antibodies were used: anti-oct4 (Thermo Fisher Scientific, Waltham, MA, USA; PA527438), anti-vasa (Abcam, Cambridge, UK; ab209710), anti-GFRA1 (Abcam, Cambridge, UK; ab84106), and anti-Nanos2 (Abcam, 161 Cambridge, UK; ab76568), which were previously successfully used as stemness markers in other fish species [19,22,34]. For this purpose, 4 μm thick sections were cut from testes fixed in 10% buffered formalin, 4% paraformaldehyde in PBS or Bouin’s solution; subsequently, de-paraffinized sections were rehydrated through graded ethanol solutions. For antigen retrieval, testis sections underwent zero to four boiling cycles, 5 min each, in citrate buffer (0.01 M, pH 6.0) in a microwave oven on high power (750 W). Endogenous peroxidase was inhibited by treating sections for 10 min with 3% H_2_O_2_ and then rinsing them with distilled water and PBS (0.01 M, pH 7.4, containing 0.15 M NaCl). Subsequently, sections were incubated for 30 min in normal horse serum (Vector Laboratories, Inc., Burlingame, CA, USA) to block nonspecific binding sites for immunoglobulins. Sections were incubated with primary antibodies diluted in PBS containing 0.1% bovine serum albumin (BSA) (Sigma-Aldrich S.r.l., Milan, Italy) as follows: anti-oct4, 1:10, 1:50 and 1:200; anti-vasa, 1:500, 1:1000 and 1:2000; anti-GFRA1, 1:10, 1:50, 1:200; anti-Nanos2, 1:40, 1:200, 1:1000. All primary antibody dilutions were tested both overnight at 4 °C and at 37 °C for 60 min. After rinsing for 10 min in PBS, immunohistochemical visualization was conducted using the Vectastain Universal Elite Kit (Vector Laboratories, Inc., Burlingame, CA, USA). This method uses the avidin–biotin–peroxidase complex procedure. Peroxidase activity was visualized by incubating for 10 min with a Vector DAB (3,3′-diaminobenzidine) Peroxidase Substrate Kit (Vector Laboratories, Inc., Burlingame, CA, USA), which produces a brown precipitate. To confirm the specificity of the immunoreaction, a control-staining procedure was performed by replacing the primary antibody with normal horse serum and PBS. Mayer’s haematoxylin was used as the counterstain in sections immunostained with anti-vasa antibodies.

### 2.4. Meagre Spermatogonial Stem Cell Isolation

In both trials, isolation of meagre SSCs was carried out using the protocol reported for roho labeo *Labeo rohita* by Panda et al. (2011) [35].

For the procedures described in the present paragraph, if not otherwise specified, Sigma-Aldrich (Milan, Italy) reagents were used. For SSC isolation, six small pieces (around 50 mg each) were excised aseptically from the testes and washed in PBS containing 2% penicillin and 2% streptomycin. Tissue samples were then washed with PBS and immersed in L-15 Leibovitz Medium where they were further fragmented with a scalpel. Subsequently, testis fragments were dissociated with type IA collagenase in L-15/DMEM-F12 medium containing 0.5% BSA, 20 mM HEPES, and 5% fetal bovine serum (FBS; Gibco, Thermo Fisher Scientific) at 28 °C and 5% CO_2_ for 2h by repeated pipetting at every 20 min interval. The cell suspension was then diluted three times with the same medium. After removal of the undissociated fragments, the suspension was centrifuged at 300× *g* for 10 min. The pellet containing germ cells was suspended in 4 mL buffer containing PBS, 2 mM EDTA, and 0.5% BSA for gradient separation. The Ficoll–Paque PLUS (GE Healthcare Life Sciences, Uppasala, Sweden) gradient was built up in a 15 mL centrifuge tube (Becton Dickinson Falcon, FranklinLakes, NJ, USA). Briefly, 3 mL Ficoll–Paque PLUS was added to the tube followed by layering of 4 mL cell suspension. The tube was then centrifuged at 800× *g* for 40 min at room temperature to ensure stratification of the different cell types. The bands obtained after centrifugation were collected in different vials using Pasteur pipettes, and the isolation of the SSC population was confirmed after observation under a phase contrast microscope. The meagre SSCs were counted after staining with a Trypan blue dye exclusion test using a Bürker chamber. Subsequently, cell aliquots were subjected to immunofluorescence analysis and in vitro proliferation experiments.

### 2.5. Meagre Spermatogonial Stem Cell Culture

For in vitro proliferation of meagre SSCs during Trial 1, two culture media were tested: the culture medium used by Panda et al. (2011) [35] for roho labeo (hereafter referred to as Medium A) and the culture medium used by Lacerda et al. (2013) [22] for Nile tilapia *Oreochromis niloticus* (referred to as Medium B). Changes made to Lacerda et al.’s (2013) [22] culture medium included (i) removal of KnockOut Serum Replacement, tilapia embryo extract, human recombinant basic fibroblast growth factor, and human recombinant epidermal growth factor and (ii) addition of 40 ng/mL rat-glial-cell line-derived neurotrophic factor (GDNF). For both stem cell cultures, fish serum previously obtained from centrifugation (1300× *g* for 10 min) of European seabass *Dicentrarchus labrax* blood taken from commercially farmed specimens was used; the serum was incubated at 56 °C for 2 h in order to inhibit complement system activity, and stored at −20 °C until it was added to the culture medium at the dilution of 1:100 v/v.

Both culture media proved to be effective at stimulating spermatogonial stem cell proliferation, so only Medium B was used for Trial 2.

To evaluate cell characteristics in culture, multi-well tissue culture plates were employed in order to provide multiple samples of the same preparation for examination at several time points after initiation of culture. Cultured cells were analyzed under phase contrast and differential interference contrast (DIC) microscopy. For proliferation and characterization studies, SSCs were seeded on 0.2% cold-water-fish-skin-gelatin-coated dishes (Sigma Aldrich, Milan Italy), at a density of 1 × 10^5^ cells/well (21 mm^2^/well) for Trial 1 and 5 × 10^4^ cells/well (35 mm^2^/well) for Trial 2, and incubated at 28 °C in an atmosphere of 5% CO_2_.

Twice per week, 50% of the cell culture medium was removed and replaced with new medium. Cell viability was assessed every 3-4 days for Trial 1 and every 7 days for Trial 2, by determining plasma membrane integrity via the Trypan blue dye exclusion test. Cell viability was expressed as percentage of viable/total cells [36]. SSC in vitro growth rate was evaluated as number of viable cells counted using a Bürker chamber. Viability and cell growth values are expressed as mean ± SD of three independent cell counts.

### 2.6. Meagre Spermatogonial Stem Cell Cryopreservation

Standard medium supplemented with 10% (v/v) FBS and 10% (v/v) dimethyl sulfoxide (DMSO, Sigma D-5879) was used to cryopreserve meagre SSCs immediately after isolation. Each cryovial was filled with 1 mL of cryopreservation medium containing 1.5 × 10^5^ cells/mL. Three samples were stored at −80 °C. After three months, the cryovials were thawed and SSCs were evaluated for viability.

### 2.7. Immunofluorescence

The immunofluorescence analysis was performed on meagre SSCs: (i) immediately after isolation for both the trials; (ii) every 3–4 days for Trial 1; (iii) and every 7 days for Trial 2, until Day 14. For this purpose, cells were centrifuged at 300× *g* for 5 min at room temperature, and then the pellet was directly fixed with 4% paraformaldehyde in culture dishes for 20 min at room temperature (RT). After rinsing with PBS containing 0.1% BSA, cells were incubated in a blocking solution containing PBS, 5% BSA, and 0.05% Triton-X-100 for 30 min at RT. Subsequently, cells were rinsed with PBS containing 0.1% BSA (three times), and then incubated overnight at 4 °C with the same antibodies used for immunohistochemistry: anti-oct4, 1:400; anti-vasa, 1:100; anti-GFRA1, 1:200; anti-Nanos2, 1:40. After rinsing with PBS containing 0.1% BSA, cells were incubated 1 h at room temperature for fluorescent detection with goat anti-rabbit IgG (H+L) secondary antibody, FITC (Termo Fisher Scientific, Waltham, MA USA, 1:500). After rinsing in PBS containing 0.1% BSA, cells were mounted on slides using Hoechst 33258 in 3:1 of glycerol to PBS solution to counterstain cell nuclei. Evaluation of cell morphology was performed by phase contrast microscopy (Nikon Eclipse, Ti-U, 400× magnification), whereas nuclear chromatin was analyzed under epifluorescence microscopy by using a Nikon Eclipse 600 microscope (400× magnification). FITC staining was evaluated by confocal microscopy by using a Nikon C1 confocal microscope (Nikon Instruments Europe, Amsterdam, The Netherlands) (630× magnification) with the aid of the EZ-C1 Gold Version 3.70 image analysis software.

### 2.8. Statistical Analysis

Meagre SSC viability was compared by chi-square test between two consecutive time intervals for Medium A, two consecutive time intervals for Medium B, and at the same time intervals for Medium A vs. Medium B. Differences in the number of viable meagre SSCs were evaluated via two-tailed Student’s *t*-test for the same groups.

Statistical analyses were performed using MS Office Excel 365, and statistical significance was accepted for *p* < 0.05.

## 3. Results

### 3.1. Histological and Immunohistochemical Analyses

**Figure 1:**
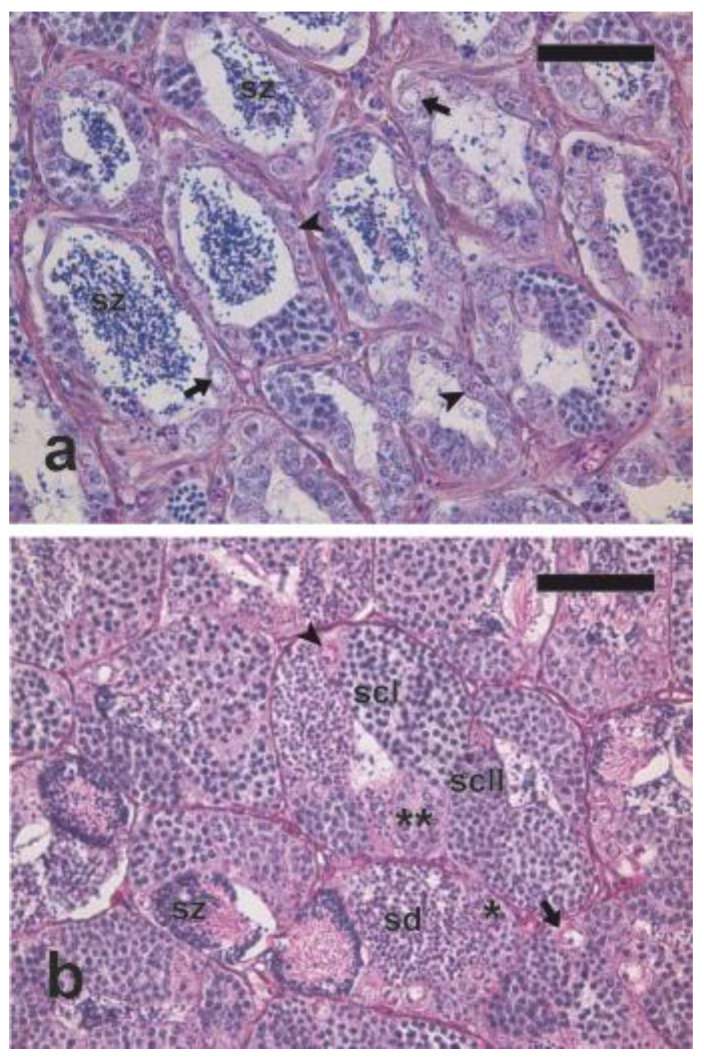
Micrographs of testis sections of meagre sampled in two periods of the reproductive cycle. (**a**) Testis section from a fish in post-reproductive state (mid-July 2017, Trial 1). The germinal epithelium is mainly constituted by spermatogonia and residual spermatozoa are visible in the lumen of the seminiferous lobules. (**b**) Testis section from a fish in active spermatogenesis (mid-March 2018, Trial 2) showing all stages of spermatogenesis. Haematoxylin–eosin (H-E) staining. Magnification bars = 50 µm. Arrows: large single A spermatogonia; arrowheads: small single A spermatogonia; asterisk: Type A spermatogonial cyst; double asterisk: Type B spermatogonial cyst; sd: spermatid cyst; scI: primary spermatocyte cyst; scII: secondary spermatocyte cyst; sz: spermatozoa.

The testes of the two fish sampled for Trial 1 had almost ceased their spermatogenic activity. Their seminiferous lobules were 94.2 ± 7.9 μm in diameter, and they showed a germinal epithelium mainly constituted by spermatogonia and had a moderate amount of residual luminal spermatozoa (Figure 1a).

The testes of the two fish sampled for Trial 2 were in active spermatogenesis; their seminiferous lobules had a mean diameter of 77.9 ± 5.0 μm and showed all stages of spermatogenesis and rare luminal spermatozoa (Figure 1b). Two types of single A spermatogonia were identified: (a) larger cells 14.2 ± 0.2 µm in diameter, with a large acidophilic cytoplasm and a roundish/ovoidal nucleus 10.6 ± 1.3 µm in diameter with a prevalent euchromatic appearance, sparse patches of heterochromatin, and a preeminent nucleolus; (b) smaller cells (mean diameter 11.0 ± 0.1 µm) with a thinner acidophilic cytoplasm and a spherical nucleus (mean diameter 7.2 ± 0.9 µm), heterochromatin dots, and one or two nucleoli. The following other germ cell types were also observed: two types of spermatogonia contained in cysts, a larger type (presumptively Type A spermatogonia; mean diameter 7.2 ± 0.1 µm) making up small cysts containing few cells, and smaller cells (presumptively Type B spermatogonia; mean diameter: 5.0 ± 0.1 µm), making up larger cysts; primary (mean diameter 4.8 ± 0.1 µm) and secondary (mean diameter 2.8 ± 0.1 µm) spermatocytes; spermatids (mean diameter of 1.7 ± 0.1 µm); flagellated spermatozoa mainly within cysts or, less frequently, in the lumen of seminiferous lobules.

**Figure 2:**
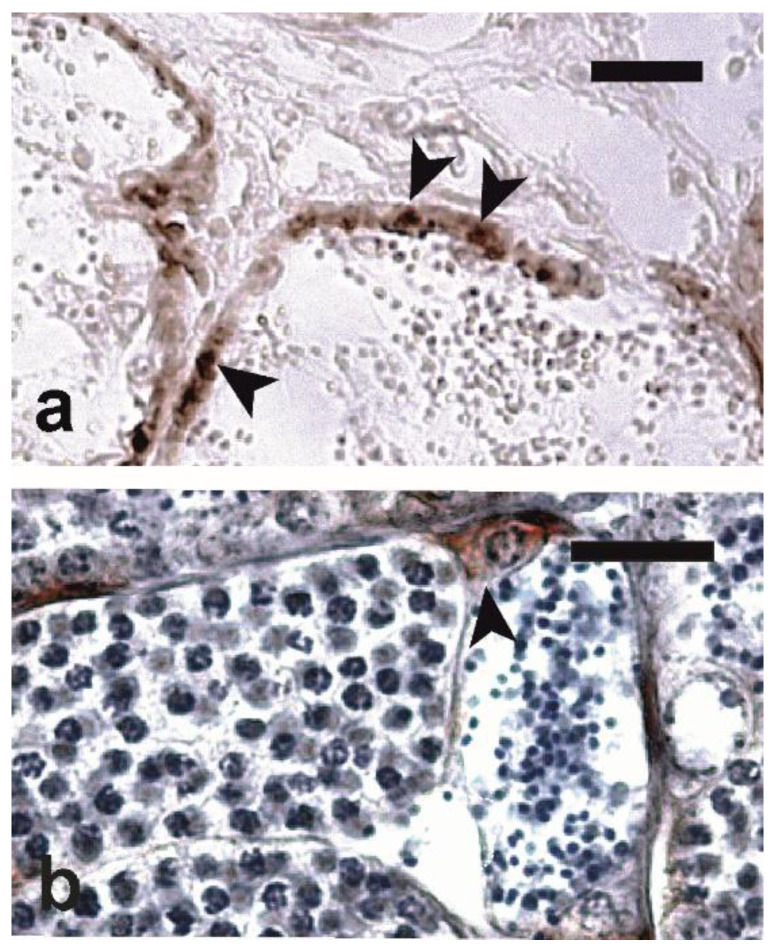
Micrographs of testis sections of meagre immunostained with (**a**) anti-oct4 antibodies (Trial 1) and (**b**) anti-vasa antibodies (Trial 2). Intralobular positive cells (arrowheads) are stained in brown. In (**b**), Mayer’s haematoxylin was used as a nuclear counterstain. Magnification bars = 20 µm.

Among the four used antibodies, only anti-oct4 and anti-vasa, in Bouin’s solution-fixed samples, successfully labeled single intralobular cells (Figure 2a,b) located throughout the length of the seminiferous lobules, with a higher density at their blinded end.

On the basis of their diameter and preferential topography, the anti-oct4- and anti-vasa-positive intralobular cells likely corresponded to the smaller single Type A spermatogonia identified on H-E sections. Anti-oct4 antibodies proved to be more effective for specifically labeling single undifferentiated Type A spermatogonia at the dilution of 1:10 and incubation at 37 °C for 60 min and after antigen retrieval with two boiling cycles of 5 min; anti-vasa antibodies were more effective at the dilution of 1:2000, with no antigen retrieval and overnight incubation at 4 °C.

### 3.2. Meagre Spermatogonial Stem Cells’ Isolation Culture and Cryopreservation

**Figure 3:**
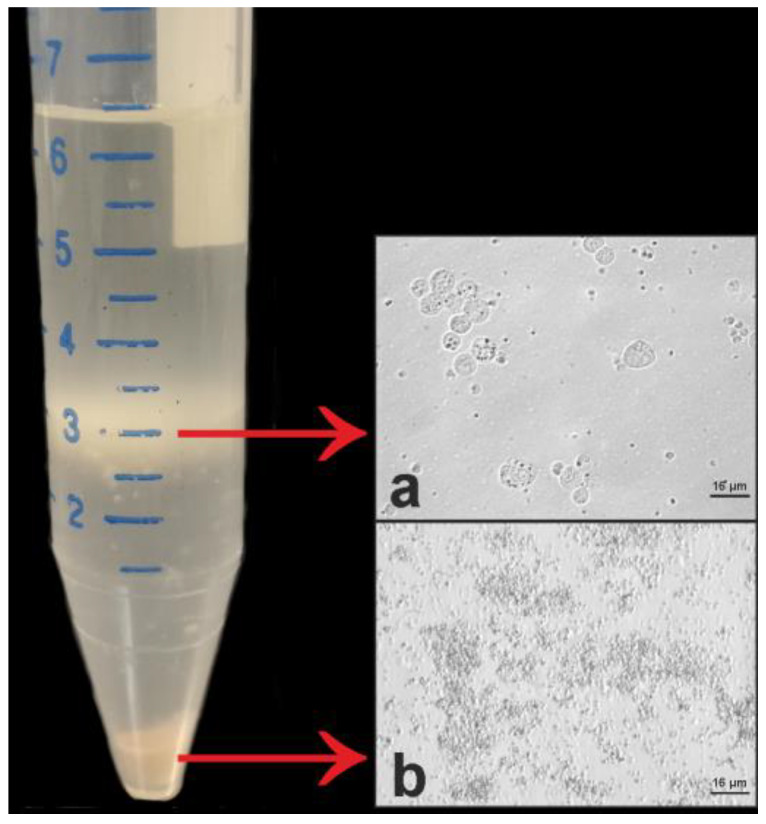
Isolation of meagre testicular cells by Ficoll–Paque PLUS gradient. The band (**a**) contained different cell types, including cells with size and appearance compatible with those of spermatogonial stem cells (SSCs). Cell debris together with putative spermatozoa and red blood cells were present in the pellet (**b**).

The results of the cell suspension centrifugation in Ficoll–Paque PLUS density gradient are shown in Figure 3. The band corresponding to the 3 mL gradation (Figure 3a) contained several cell types, two of which showed morphology and size corresponding to the two types of single A spermatogonia identified in the H-E sections.

After the density gradient centrifugation, from a total of 400 mg tissue, 7.9 × 10^6^ cells and 1.7 × 10^6^ cells were obtained in Trials 1 and 2, respectively. In both trials, soon after cell collection, cell viability was found to be close to 100%.

**Figure 4:**
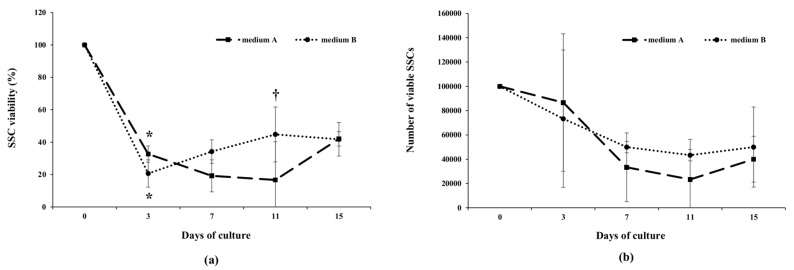
Viability (**a**) and proliferation rate (**b**) of meagre spermatogonial stem cells (SSCs) cultured in vitro in Medium A (Panda et al., 2011 [35]) or Medium B (modified by Lacerda et al., 2013 [22]) (Trial 1). * indicates significant differences in cell viability versus the preceding evaluation; † indicates significant difference in cell viability between the two media at Day 11 (chi-square test, *p* < 0.05). No statistical differences were observed in the proliferation rate between the two culture media (Student’s *t*-test; *p* < 0.05).

The behavior of cells in the two culture media during Trial 1 was very similar (Figure 4), with the exception of a significantly higher SSC viability in Medium B after 11 days of culture (Figure 4a). A decreased number of viable cells from the day of seeding (Day 0) until Day 7 was observed (Figure 4b). It was followed by a stationary phase in which cell numbers were stable until Day 15. Both cell cultures were stopped after 15 days due to the accumulation of debris in culture media.

**Figure 5:**
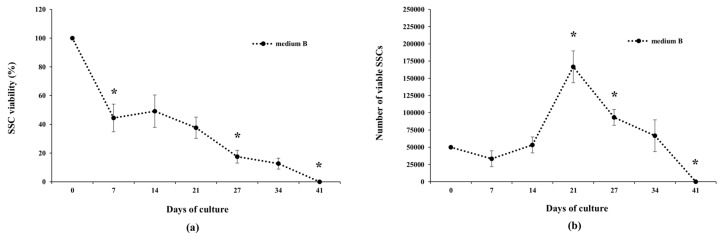
Viability (**a**) and proliferation rate (**b**) of meagre spermatogonial stem cells (SSCs) cultured in vitro in Medium B (modified by Lacerda et al., 2013 [22]) (Trial 2). * indicates significant difference in cell viability (chi-square test; *p* < 0.05) or proliferation rate (Student’s *t*-test; *p* < 0.05) versus the preceding evaluation.

The results of Trial 2 showed that meagre SSCs could be cultured up to 41 days. In fact, although cell viability showed a progressive decrease during long-term in vitro culture (Figure 5a), SSCs showed active proliferation from Day 7 to Day 21 (exponential phase) and declined thereafter (Figure 5b).

About 20 days after the beginning of the trials, cultured meagre SSCs showed a tendency to form clusters that continued to proliferate, forming colonies lightly attached at the bottom of the well.

Cell-freezing experiments revealed that meagre SSCs could be stored in DMSO-based cryopreservation medium at −80 °C. After thawing, cell viability was checked and found to be around 28.5%. In detail, 44.4 × 10^4^ viable cells were identified from a total of 155 × 10^4^ cells. No differences in cell morphology were identified after freezing/thawing.

### 3.3. Immunofluorescence Analysis

**Figure 6:**
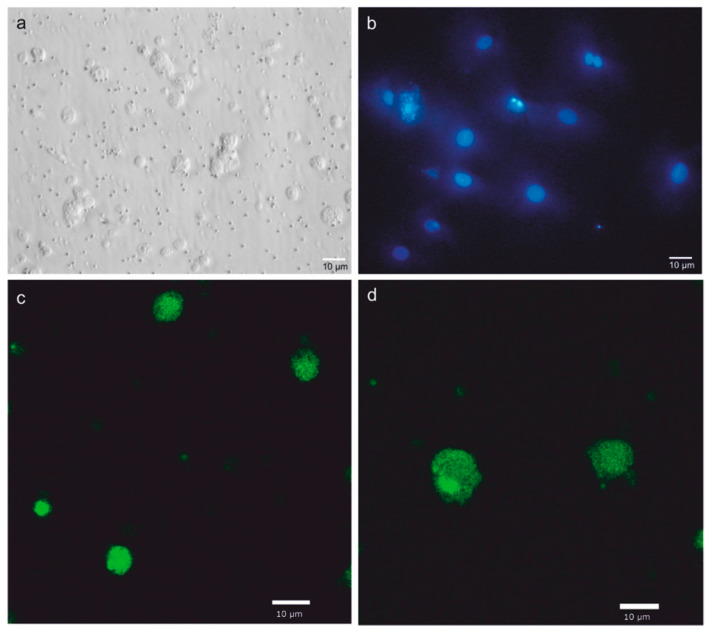
Characterization of meagre spermatogonial stem cells (SSCs) cultured in vitro. Photomicrographs of cells exhibiting round-shape morphology, as observed via phase contrast microscopy (**a**), and presence of nuclei and small cytoplasm, as observed via epifluorescence microscopy after Hoechst staining (**b**). Confocal microscopy evaluation revealed anti-oct4- (**c**) and anti-vasa-positive (**d**) cells after 14 days of in vitro culture.

The results of the immunofluorescence analysis showed that oct4 and vasa were expressed in meagre SSCs (Figure 6). The epifluorescence analysis revealed the presence of nucleated cells with small volumes of cytoplasm (Figure 6b). Confocal microscopy evaluation revealed that oct4-positive cells had nuclear diameters ranging between 7 and 9 µm, and vasa-positive cells were between 10 and 11 µm. Interestingly, oct4 (Figure 6c) and vasa (Figure 6d) were still expressed in SSCs after 14 days of culture. No signals were detected when using GFRA1 and Nanos2 antibodies.

## 4. Discussion

Meagre spermatogonial stem cell isolation, characterization, and in vitro proliferation are necessary steps for the establishment of a xenotransplantation technology that might support the development of a self-sustained, large-scale aquaculture for this species. In fact, despite the market appreciation, meagre aquaculture production in Europe is limited by broodstock availability and difficulty managing reproduction in captivity [7,13,15].

In the present study, meagre SSCs were isolated, characterized, and cultured in vitro, taking advantage of experimental protocols developed for other fish species, i.e., roho labeo [35] and Nile tilapia [22]. In particular, efforts were made to simplify the reported laboratory procedures in order to make SSC isolation and in vitro proliferation less expensive and time-consuming.

All four hatchery-produced males used for the two trials reported in the present study were adults, based on the size at sexual maturity reported for the wild population from the Mediterranean Sea [10]. In adult fish, spermatogonial stem cell self-renewal is correlated to the phase of the reproductive cycle and shows two peaks, one at the beginning and one at the end of the reproductive season [37,38,39]. In order to optimize the procedure for stem spermatogonia isolation, the two trials were carried out in mid-March and in mid-July, when wild meagre from the Mediterranean have been reported to be in early gametogenesis and in the spawning/postspawning stage, respectively [10].

Meagre spermatogonia were first identified on H-E-stained testis sections based on descriptions reported for other fish species [32,39]. In the germinal epithelium, two types of single (i.e., not making part of spermatocysts) germ cells were observed: (a) smaller cells, around 11.0 µm in diameter, with a spherical nucleus containing one or two nucleoli; (b) larger cells, around 14.0 µm in diameter, with a roundish/ovoidal euchromatic nucleus containing a preeminent nucleolus. These two cell types likely correspond to the two Type A undifferentiated spermatogonia (A_und_* and A_und_) of Schulz et al.’s (2010) [39] classification, the former being stem spermatogonia, responsible for germ cell self-renewal; and the latter being Type A undifferentiated spermatogonia, the activity of which is related to differentiation and rapid proliferation toward meiosis.

In order to characterize meagre SSCs, antibodies against four stemness markers used for the identification of germ cells in fish species were selected: oct4, vasa, GFRA1, and Nanos2. Oct4, the octamer-binding transcription factor 4 encoded by the gene *POU5F1* [40,41], has been detected in Type A undifferentiated spermatogonia of Japanese flounder *Paralichthys olivaceus* [42], medaka *Oryzias latipes* [43,44,45], roho labeo [35], Nile tilapia [22], and dogfish *Scyliorhinus canicula* [46], as well as in primordial germ cells (PGCs) and pluripotent embryo stem cells of Japanese flounder [42] and medaka [43,44,45]. Vasa is a highly conserved member of the DEAD box protein family of putative RNA helicases [47], and it is expressed in PGCs of zebrafish *Danio rerio* [48,49,50,51,52], rainbow trout *Oncorhynchus mykiss* [53,54], Nile tilapia [55], and sturgeons [56], as well as in undifferentiated Type A spermatogonia of zebrafish [57,58], rainbow trout [59,60], Nile tilapia [22,61], medaka [62], roho labeo [35], Japanese amberjack *Seriola quinqueradiata* [63], tambaqui *Colossoma macropomum* [64], and salmonids [65]. The glycosyl phosphatidylinositol-linked ligand-binding subunit GFRA1, a component of the receptor complex binding the glial-cell-line derived neurotrophic factor (GDNF) [30,66,67] has been found in Type A undifferentiated spermatogonia of Nile tilapia [22], rainbow trout [67], and dogfish [46]. The germ-cell-intrinsic factor Nanos2 is a key regulator for the maintenance and modulation of SSC self-renewal [30,68,69,70], and its expression is believed to be induced or maintained by the GDNF/GFRA1 signaling pathway [71]. Nanos2 is expressed in zebrafish PGCs [72] and in undifferentiated Type A spermatogonia of rainbow trout [73] and Nile tilapia [22].

Both the immunostainings (immunohistochemistry and immunofluorescence) carried out in the present study showed that the smaller Type A spermatogonia expressed oct4 and vasa, whereas the larger Type A spermatogonia did not immunoreact with any of tested stemness markers, thus confirming the identification of the two cell types proposed on the basis of the basic histological staining. This was in agreement with our previous observation on greater amberjack showing that only smaller Type A spermatogonia immunoreacted with anti-oct4 antibodies [32]. The stem nature of the isolated SSCs was confirmed by immunofluorescence until 14 days of culture, and this result was coherent with literature data [22,30,35].

In the present study, neither GFRA1 nor Nanos2 labeling were observed in meagre testis sections. Similar results were reported for roho labeo, the spermatogonia of which did not express GFRA1, possibly due to interspecific variability among fishes [35]. The absence of positivity to Nanos2 in the present study could be strictly correlated to that of GFRA1, since GDNF/GFRA1 signaling is essential for maintaining Nanos2 expression [71].

Anti-oct4- and anti-vasa-positive cells were distributed along the seminiferous lobules in all the sampled testes, although these cells were more abundant at the blind ends of the lobules. In particular, the testes sampled in mid-July had a germinal epithelium mainly constituted of anti-oct4- and anti-vasa-positive spermatogonia. In fish species with seasonal reproductive cycles, at the end of the spawning phase, when the spermatogenic activity is arrested and the lumina of seminiferous lobules are emptied, the germinal epithelium tends to appear as a simple cuboidal epithelium consisting mainly of spermatogonia [37,38,74,75]. In swordfish [74] and Atlantic bluefin tuna [75] sampled in this phase of the reproductive cycle, single spermatogonia were strongly immunopositive to anti-proliferating-cell nuclear antigen and, therefore, they were in active proliferation. Therefore, the results of the present study support the hypothesis that proliferating single spermatogonia found in the germinal epithelium at the end of the reproductive season are actually SSCs, divisions of which are aimed at restoring the germ cell population needed for the following reproductive cycle [38,76].

Meagre SSC isolation was carried out by following the protocol reported for roho labeo by Panda et al. (2011) [35]. The methodology reported by Panda et al. (2011) [35] consists in two steps of SSC purification: (1) use of the Ficoll–Paque PLUS gradient to separate the different cell types of the digested testicular tissue; (2) use of the magnetic-activated cell sorting method to enrich the pool of roho labeo SSCs. In the present study, only the first step was applied to separate the different cell types of the meagre testicular tissue by Ficoll–Paque PLUS. Differently from Panda et al. (2011) [35] in which two cell layers were obtained, the gradient density centrifugation carried out for meagre SSC isolation led a single distinct band corresponding to the 3 mL gradation tube. In this distinct band, several cell types were observed, including the two types of single A spermatogonia identified in the histological analysis. Trial 1 allowed the isolation of a higher number of immature germ cells compared with Trial 2. This might have been due to the presence of a moderate amount of spermatozoa in the lobule lumina of the testes used in the Trial 1, and suggests the use of fish in the early gametogenesis stage rather than fish in spawning/postspawning condition in order to maximize stem cell yield.

Magnetic-activated cell sorting [35] or cytofluorimetry [77] would have allowed further purification of the targeted cells. However, successful transplantation was obtained in Nile tilapia by using a pool of spermatogonia also including cells negative to stem markers [22]. The indication of the optimal phase of the reproductive cycle (early gametogenesis) provided in the present study could be an effective tool for obtaining good amounts of SSCs that might avert the need for a complex and expensive procedure difficult to apply in a fish farm.

The proliferative rate of the isolated meagre SSCs was tested using two culture media standardized for roho labeo [35] and Nile tilapia [22]. Indeed, in the present study, the culture medium reported by Lacerda et al. (2013) [22] was simplified by substituting human recombinant basic fibroblast and human recombinant epidermal growth factors with GDNF. In Trial 1, both tested culture media were effective in stimulating the proliferation of meagre SSC up to 15 days without significant differences in the number of proliferating cells or in their viability, and the interruption of the trial was due to the accumulation of cell debris in the media. The accumulation of debris might have occurred due to the presence of differentiated germ cells or somatic cells in the distinct band isolated with Ficoll–Paque PLUS from spawning/postspawning fish. In Trial 2, when only the modified Lacerda et al. (2013) [22] culture medium was used, better results were obtained in terms of meagre SSC viability and proliferation, and the cell cultures were prolonged to 41 days. Possible reasons for the better results obtained in Trial 2 could include (a) improvement of technical procedures including the higher frequency of culture medium replacement; (b) the use of GDNF as growth factor, which was reported to be very effective in stimulating in vitro proliferation of zebrafish spermatogonial cells [78]. Regarding the use of GDNF in fish SSC culture medium, although Lacerda et al. (2013) [22] did not include it in the formulation for Nile tilapia SSC in vitro proliferation, their cultured cells expressed its receptor, GFRA1. In the present study, SSCs did not apparently express GFRA1 despite the presence of its ligand GDNF in the culture medium. It is possible to hypothesize that the dose of GDNF used in our culture media was not sufficient to stimulate the in vitro expression of GFRA1; however, since we did not observe anti-GFRA1 immunoreactivity in chemically-fixed, paraffin-embedded sections, we cannot exclude the lack of specificity of the antibodies used in the present study towards meagre GFRA1.

Cryopreservation of fish SSCs and their transplantation represent highly innovative tools for the conservation of genetic stocks of valuable animals or endangered species [20,21]. Cryopreserved Nile tilapia SSCs were able to survive and proliferate in culture and, after transplantation, to colonize testes, generating cysts in 88% of the recipient fish [79]. In the present study, meagre SSCs kept their viability after thawing, although at a quite low percentage (about 29%). For this purpose, a commonly used concentration of DMSO (10%) was used [79], corresponding to 1.4 M. Our data can be compared with those of a recent study by Marinović et al. (2019) [80], in which zebrafish spermatogonia frozen either with 1.0 or 1.6 M DMSO showed cell viability values around 20% or 15%, respectively. However, other cryoprotectants or other DMSO concentrations might improve spermatogonia viability after thawing, since it has been shown that small differences in DMSO concentrations may remarkably affect this parameter [80].

In conclusion, in the present work, we identified meagre spermatogonial stem cells via basic histology, immunohistochemistry, and immunofluorescence and isolated them after digestion of testis samples. The isolated stem spermatogonia were cultured in vitro, showing a good proliferating capacity until 41 days of culture. A first attempt to cryopreserve stem spermatogonia was carried out, showing encouraging results. The results of the present study represent the basis for the development of a germ cell xenotransplantation technology that might support the development of the aquaculture production of this species on a large scale.
